# *The EPMA Journal* introduces a new type of research article dedicated to predictive, preventive and personalised medicine

**DOI:** 10.1186/1878-5085-4-11

**Published:** 2013-03-28

**Authors:** Olga Golubnitschaja

**Affiliations:** 1 European Association for Predictive, Preventive & Personalised Medicine, Brussels, Belgium; 2 Department of Radiology, Rheinische Friedrich-Wilhelms University of Bonn, Sigmund-Freud-Str. 25, Bonn 53105, Germany

## What are the purpose and expected impacts of this action?

The Editorial Board and BioMed Central team have been working together to develop a journal-specific outline for research articles suitable for publication in *The EPMA Journal*[1]. The purpose of this action is to promote scientific excellence in the area of predictive, preventive and personalised medicine (PPPM), through a better understanding of the integrative approach used in PPPM, innovation in biomedical technologies and comprehensive interpretation of the achieved results for accelerated adoption of the top sciences into daily medical practice and advanced health care focused on patient needs.

## Points to consider when writing a research manuscript

When preparing a research manuscript for *The EPMA Journal*, authors should consider the following aspects:

1. The research topic must be in keeping with the aims and scope of the journal [2], i.e. dedicated to predictive diagnostics, targeted preventive measures and treatment algorithms tailored to the person. The authors should consider multifunctional, experimental and technological approaches and result interpretations in terms of PPPM.

2. A research manuscript should be dedicated to the development of PPPM-related technologies and demonstrate an innovation that might be presented by hybrid technologies (e.g. complementary imaging), a reasonable combination of diagnostic approaches (e.g. imaging in combination with omics, complementary levels of molecular detection), advanced therapy targeting and innovative drug delivery systems, which present real progress beyond the state of the art in diagnostics, patient stratification and personalisation of treatments.

3. The manuscript should be presented to the highest didactic level. *The EPMA Journal* focuses on an interdisciplinary and multidisciplinary approach in educating professionals. The reason for this is that the PPPM objectives can only be achieved through a close collaboration between several professional groups. Consequently, even the most sophisticated approaches and technologies should be described in a way that is understandable to young professionals and specialists active in other biomedical fields outside of the author’s area of expertise. For this reason, it is highly recommended to make use of high-quality illustration materials, such as images and graphs, to introduce innovative technologies, new approaches, proposed molecular, subcellular and cellular mechanisms, etc.

## Why are there specific structural features required for research manuscripts in *The EPMA Journal*?

*The EPMA Journal* does not aim to compete with or duplicate the work of other journals dedicated to individual types of technologies, discovery of single biomarkers, drug targeting, medical imaging and so on. The journal promotes the use and amalgamation of knowledge collected in individual scientific and technological branches in order to create integrative PPPM-related approaches that have potentials to advance health care. Therefore, any scientific and technological innovation presented in research manuscripts submitted to *The EPMA Journal* should consider the context of PPPM integration and the advancement of medical services. Consequently, the following article sections are asked to be included and these points should be considered prior to submission of a manuscript:

• Introductory overview of the topic: this section should demonstrate the relevance of the article for the integrative field of PPPM. A detailed field overview with citations to all relevant literature is requested to inform the reader of the state of the art and demonstrate current deficits relevant to health care quality.

• A detailed analysis of the achieved results is an obligatory section of the manuscript to demonstrate the robustness of the discovery.

• An interpretation of the discovery should be provided in the context of PPPM followed by the spectrum of functions proposed for its potential utilisation in health care.

• Expert recommendations are requested to outline perspectives for concomitant developments in the field and related branches.

The Editorial Board is confident that our current initiative will become well understood and strongly supported by the journal readers and authors.
